# An Integrative Behavioral Couple Therapy (IBCT)-Based Conflict Prevention Program: A Pre-Pilot Study with Non-Clinical Couples

**DOI:** 10.3390/ijerph18199981

**Published:** 2021-09-23

**Authors:** Jorge Barraca, Elvira Nieto, Thomas Polanski

**Affiliations:** 1 Department of Psychology, Universidad Camilo José Cela, Calle Castillo de Alarcón 49, Urbanización Villafranca del Castillo, 28692 Madrid, Spain; 2 Private Practice, Calle Goya 83–3º Derecha, 28001 Madrid, Spain; elviranietopsicologia@gmail.com; 3 Private Practice, Mariano Paredes N70-122 and Moisés Luna Andrade, Quito 170303, Ecuador; polanskij@gmail.com

**Keywords:** couple conflict, couple therapy, Integrative Behavioral Couple Therapy, prevention programs

## Abstract

Integrative Behavioral Couple Therapy (IBCT) has demonstrated its efficacy treating severe couple conflict. Nevertheless, its capacity to prevent such conflicts before they appear has not been analyzed. The following empirical study examines the effectiveness of a conflict prevention program based on IBCT’s main therapeutic strategies (empathic joining, unified detachment). A sample of 12 individuals (six couples) from the Community of Madrid completed the *DAS* (Spanier, 1976; Martín-Lanas et al., 2017), *IBCTQ* (Barraca et al., 2017), and *ASPA-A* (Carrasco, 1996) pre-treatment, posttreatment, and at a three-year follow up. Three of these couples were randomly assigned to the experimental group, in which they received five, 120-minute sessions of an IBCT-based conflict prevention program. The three remaining couples were assigned to a control group and received no treatment. Results indicated that the experimental couples grew in their acceptance of differences and significantly improved their level of empathic joining and unified detachment; they also manifested greater satisfaction in their total *DAS* score. At the three-year follow up, neither group showed significant changes with regard to their posttreatment scores. Although the data are based on a small number of couples and should be replicated, the results suggest that a program based on IBCT strategies can help prevent couple conflict up to three years after its application.

## 1. Introduction

Most couples seek therapy only when experiencing high levels of distress and numerous conflicts, with the corresponding risk of relationship dissolution [1,2]. This assumption—that couples should only go to therapy after presenting severe problems—is dangerous. The higher the level of conflict when the couple initiates therapy, the greater the possibility of therapeutic failure and the greater the risk of one or both partners developing symptoms of anxiety, depression, substance abuse, or other behaviors that jeopardize their health, up to and including suicide [3,4]. If generic risk factors for couple conflict were tackled earlier, their appearance could be prevented or diminished, resulting in greater relationship satisfaction and psychological well-being both in the medium and long-term. Such prevention would also alleviate the pressure on legal and health services, avoiding the personal, social, and economic costs that accompany relationship deterioration [5,6].

In spite of this, the development of couple conflict prevention programs is scarce, and their empirical evaluation shows that existing programs suffer from important problems, especially with regard to limitations in their design. A study by Christensen and Heavy [7] on the efficacy of three of the most well-known prevention programs of the 1960s, 1970s and 1980s—the *Couples Communication Program* [8], the *Relationship Enhancement Program* [9], and the *Prevention and Relationship Enhancement Program* [10,11]—found little evidence that they had medium or long-term positive effects. At the same time, the theoretical heterogeneity of these interventions, which included systemic, humanist (in the line of Carl Rogers), social learning, and mixed (learning and cognitive behavior theory) models made it difficult to glean which components or processes were responsible for the programs’ results. More specifically, while Christensen and Heavy’s work showed that these interventions can change couple behavior and produce short to medium-term improvements in communication, self-revelation, empathy, stability, and relationship adjustment [12,13,14,15,16,17], an exhaustive examination of the meta-analysis conducted by Giblin et al. [14] and Hahlweg and Markman [16] seemed to indicate that these effects dissipate with time. Upon analyzing the programs as a whole, Christensen and Heavy concluded that the *Couples Communication Program* obtained the best results in comparison with the others but that the *Prevention and Relationship Enhancement Program* was the only one that continued to show some benefit beyond six months posttreatment.

In 2004, Carroll and Doherty [18] carried out a systematic review of premarital prevention and education programs. Taking into account twelve experimental studies—again heterogeneous in theoretical orientation (systemic, social and behavioral learning, social exchange theory, and diverse psychoeducational perspectives)—results showed that these programs are generally effective in producing immediate and short-term gains in interpersonal abilities and relationship quality. Nevertheless, because of the lack of extensive follow-up research, conclusions about long-term effectiveness could not be reached. At the same time, the diversity of theoretical orientations and measurement instruments made it difficult to determine which treatment elements were useful and efficacious.

A study by Ledermann et al. [19] of their well-controlled *Couples Coping Enhancement Training* showed similar results. They randomly assigned a large sample of 100 couples to experimental and control groups. The program produced positive effects immediately after completion, but these progressively disappeared at six-month and one-year follow ups. 

More recently, Rogge et al. [20] tested their *Compassionate and Accepting Relationships through Empathy (CARE)* program against the aforementioned *Prevention and Relationship Enhancement Program*. *CARE* is designed to strengthen relationships by teaching couples abilities to better empathize with and support one another. These abilities are partially based on aspects of Integrative Behavioral Couple Therapy (IBCT) [21] with components of empathic joining, expressing soft emotions, acceptance, perspective change, and psychological distancing. In addition to forming part of either of these programs, couples could also be randomly assigned to a group that received one session of instruction on the importance of being more aware of the relationship or to a control group. All of the 174 participating couples were engaged or recently married and were not experiencing significant stress in their relationship. Results showed that at the end of the training, there were no significant differences among the three experimental groups, but all fared better than the control group, which had a higher probability of relationship dissolution within the following three years. In order to explain the lack of differences among the experimental groups’ results, the authors hypothesized that the programs did not have sufficiently distinct components, that the measurement instruments were not ideal (and had only consisted of self-completed questionnaires), or that the sample was not sufficiently heterogeneous with respect to economic situation or relationship status (engaged and recently married couples).

Another more recent marriage prevention program is the *Marriage Check-up* [22]. Based on both motivational interviewing and IBCT principles, it consists of an assessment session and a feedback session; the first lasts three to four hours and the second lasts around two hours. A pilot study (*n* = 29) showed that positive effects lasted two years after application; however, there was no control group with which to compare results, and the sample may have contained a mix of distressed and non-distressed couples [23]. Most subsequent studies such as Cordova et al., Trillingsgaard et al., and Gordon et al. [24,25,26] also show positive effects, and benefit from the use of control groups and larger samples. However, they only collect short-term follow-up data. An exception is the study of Cordova et al. (*n* = 215) [27] on annual marriage check-ups, which included a second marriage check-up one year after the first and data collection at a two-year follow-up. Treatment couples showed significant gains in relationship satisfaction, intimacy, and acceptance after the first check-up, usually with an initial spike followed by a tapering effect that was still significantly better at the one-year follow up than at pretreatment levels. The second marriage check-up seemed to help maintain these gains for the two-year follow-up, again contributing to an initial spike in all variables followed by a tapering effect. Unfortunately, results were not disaggregated for distressed and non-distressed couples, and the mix of motivational interviewing and IBCT-based components make it difficult to determine which treatment elements were efficacious.

A small aside must also be made to recognize efforts by Andrew Christensen and Brian Doss to make an IBCT program available to a wide variety of couples through their online adaptation of IBCT therapy called *Our Relationship Program*. While the program’s design seems as though it could be easily adapted for use with non-distressed couples, the only published data on its effectiveness pertains to its use with distressed couples [28,29]. 

The objective of the current investigation is to begin to correct some of the limitations of earlier studies by creating a conflict prevention program that: (1) is based on a single theoretically and experimentally sound model; (2) possesses stricter and better-defined content to help identify effective prevention components, and (3) demonstrates potential to generate significant long-term positive effects. Pilot studies are generally carried out to test whether interventions merit further investigation and/or to guide the design of larger, definitive trials. They can do the former by demonstrating treatment tolerance and providing initial efficacy data, and the latter by calculating population variance and recruitment and retention rates, which help to estimate the required sample size for a definitive trial [30,31]. The current investigation concentrates on the first aspect looking to demonstrate tolerance and preliminary evidence of effectiveness for an IBCT-based conflict prevention program. Given the positive results that IBCT has shown in a large, rigorous, randomized clinical trial [32,33], the development, application, and quantitative and qualitative evaluation of an IBCT-based couple conflict prevention program is considered to be of investigative and clinical interest. The current study was carried out in Spain with an urban population in the city of Madrid. While cultural differences between this population and the groups studied in other relationship prevention programs (usually couples from the United States) could influence IBCT’s effectiveness, IBCT therapy has been carried out during various decades in Spain with positive results, and we believe that its main elements (especially acceptance) are universal with regard to increasing well-being in couples, which is an opinion that seems to be shared by the therapy’s original authors who have years of experience working with multi-cultural couples [34].

## 2. Materials and Methods

### 2.1. Participants

The sample was composed of 6 couples (12 individuals) who were recruited using an ad placed on social media. The ad contained the following information: “Prevent future couple conflicts. A free, individualized course is being offered by psychology professionals for couples that want to strengthen the quality of their relationship. You can obtain more information by calling the following number: 671XXXXXXX. What does the course consist of? In this course you will learn to: (1) better understand your partner (their behaviors, thoughts and emotions); (2) communicate more effectively with them (to express what you both feel and think adequately); (3) prevent future couple conflict; and (4) nourish the strengths that unite you and your partner. How many days? 5 days (1 day a week, total: 5 weeks). How much time? 1 h and 30 min per session.” Eight couples replied to the ad, but two were discarded—one because of scheduling conflicts and the other because one of the partners expressed little commitment to the program—resulting in a 75% recruitment rate. Three of the couples were randomly assigned to the experimental group and three were assigned to the control group. Inclusion criteria required that: (1) participants be over 18 years of age, (2) neither partner have a mental disorder, (3) the couple not currently be in, nor have attended in the past, any form of couple therapy, and (4) the couple not be in the process of separating. The presence of a current mental disorder was discarded using a clinical interview, and all couples scored within a normal range for relationship satisfaction on the *Dyadic Adjustment Scale* [35,36], the properties of which are discussed in the next section. The sample’s sociodemographic characteristics are presented in Table 1.

### 2.2. Instruments

#### 2.2.1. Quantitative Measures

*Dyadic Adjustment Scale–DAS*—[35,36]. This 32-question, Likert-style measure evaluates the quality of a couple’s relationship using a global scale for dyadic adjustment and four subscales: (1) consensus; (2) satisfaction; (3) affectional expression, and (4) cohesion. The Spanish adaptation has shown good internal consistency (0.88, 0.88, 0.69, and 0.85 for each subscale), and factor analysis has demonstrated the construct validity of the instrument. The correction and interpretation of results is carried out by transforming the direct scores into T scores. 

*Integrative Behavioral Couple Therapy Questionnaire—IBCTQ* [37]. A 68-question, Likert-style instrument designed to measure the main concepts of IBCT. It includes four dimensions: (1) acceptance; (2) empathic joining; (3) unified detachment, and (4) tolerance. The higher the score in each dimension, the better the couple’s situation with regard to that variable (and the higher their resilience to conflict), according to IBCT’s theoretical model and intervention strategies. Examples of items for each dimension include “I find it positive that my partner and I are different”, “When my partner does something that hurts me, I try to express to them how I feel without attacking them”, “My partner and I try to resolve problems maintaining a united front”, and “When my partner does something that annoys me, I try to see the positive side of their behavior”. In a preliminary study with 485 subjects, interdimensional correlations were elevated and significant and the internal consistency of the subscales was adequate to good (Cronbach’s alpha of 0.88, 0.85, 0.90, and 0.61 for each subscale and 0.93 for the entire instrument). Construct validity was tested by correlating results with the *DAS*, *CSI*, *ESFA*, and *ASPA*, producing significant correlations in the expected directions. The instrument is corrected by comparing the direct scores with population scores.

*Couple Assertiveness Questionnaire* (*Cuestionario de Aserción en la Pareja (ASPA))* [38]. The *ASPA* is a two-part, Likert-style instrument with 40 questions per section. In the first section (Form A), the subjects evaluate themselves, and in the second section (Form B), they evaluate their partner’s behavior. The instrument evaluates four types of communication that are common in couples’ daily experience: (1) assertive; (2) aggressive; (3) submissive, and (4) passive aggressive. Direct scores are transformed to percentiles, and the percentage of each type of communication is calculated. The greater the percentage of assertive communication compared to the other three styles, the better the emotional situation and conflict resolution capacity of the couple. Psychometric study of the instrument was carried out with 418 couples. The instrument showed good internal consistency (Cronbach’s alpha of 0.83, 0.81, 0.75, and 0.84 respectively, with a total value of 0.89 for Forma A and 0.90 for form B). Factor analysis and correlation with the *DAS* questionnaire showed good construct validity. 

#### 2.2.2. Qualitative Measures

*Satisfaction Questionnaire (CS)*. A short, ad hoc questionnaire was designed to evaluate the participant’s satisfaction with the prevention program. It consisted of seven Likert-style items scored from 0 (very low) to 4 (very high), each measuring a different aspect of satisfaction: (1) general satisfaction regarding the individual’s expectations for the program; (2) interest in the topics covered; (3) interest in the strategies presented; (4) the possibility of applying these strategies; (5) perception that these strategies would be helpful for addressing future marital problems; (6) level of satisfaction regarding the professional who had imparted the program, and (7) recommendation of the program to other couples. This questionnaire’s psychometric properties have not been evaluated; thus, it is considered to be a qualitative measure. 

*In-session notes.* During program sessions, experimental couples’ verbalizations in relation to program content were registered as evidence of their subjective reactions to IBCT strategies and exercises.

Treatment tolerance was evaluated by taking into account both quantitative and qualitative relationship and program satisfaction measures to ensure that the program was well-accepted by couples without causing unintended iatrogenic effects. 

#### 2.2.3. Procedure

All of the subjects signed a consent form approved by the Universidad Camilo José Cela’s Ethics Committee. The study was conducted according to the guidelines of the Declaration of Helsinki and approved by the aforementioned committee (13_CEI; 26/04/2021).

The prevention program’s content was delivered in five sessions and based on diverse IBCT manuals [21,39,40] and articles [1,41,42,43]. The content of each session and program details are described in Table 2. The prevention program was designed to help in the early stages of possible couple conflict.

The aforementioned evaluation instruments were applied to the control and experimental groups before beginning the program (pre-program evaluation), at the end of the program (post-program evaluation), and three years after program completion (follow-up evaluation). 

The program was applied by the same therapist to all couples in order to guarantee a similar application and fidelity to content. The therapist was a certified psychologist with experience and a master’s degree in clinical psychology. She was an expert in IBCT, trained through courses, workshops, and direct supervision. She did not have any interests in nor receive any benefits tied to the type of results obtained. Interventions were carried out in a private office.

#### 2.2.4. Design and Data Analysis

A quasi-experimental design was used. As a result of the small sample size (*n* = 12) and the lack of normal distribution for variables, non-parametric statistical tests were used for data analysis. The Wilcoxon signed-rank test was employed to evaluate differences in pre-intervention, post-intervention and follow-up scores in the experimental and control groups (intragroup measure). The Mann–Whitney *U*-test was used to measure significant differences between the experimental and control groups at the pre-intervention, post-intervention, and follow-up measurement points (intergroup measure). One experimental couple did not reply to the three-year follow-up measures since they had separated during that period; thus, a missing data approach was incorporated into follow-up analyses. All statistical calculations were carried out using IBM’s Statistical Package for the Social Sciences (SPSS v. 21, IBM, Armonk, NY, USA).

## 3. Results

As can be observed in Table 3, the control group showed no significant change during the experimental group’s intervention period, whereas the experimental group showed significant positive increases (Wilcoxon *Z*, *p* ≤ 0.05) in their *DAS* scores for the global index of dyadic adjustment (*Z* = −1.897; *p* = 0.02) and for two subscales: dyadic satisfaction (*Z* = −2.032; *p* = 0.02) and affectional expression (*Z* = −1.761; *p* = 0.03). Near significant positive increases were found for dyadic consensus ($\bar{X}$ pre = 46.67; $\bar{X}$ post = 48.67; *Z* = −1.511; *p* = 0.06) and dyadic cohesion ($\bar{X}$ pre = 13.83; $\bar{X}$ post = 15.33; *Z* = −1.355; *p* = 0.08). The only near significant change for the control group, regarding dyadic satisfaction, was in a negative direction.

A similar pattern was observed in the *IBCTQ* data. The experimental group showed statistically significant increases in empathic joining (*Z* = −1.782; *p* = 0.03), unified detachment (*Z* = −2.201 *p* = 0.01), and their global *IBCTQ* scores post-intervention (*Z* = −1.997 *p* = 0.02). Positive changes in acceptance and tolerance were near significant (*Z* = −1.363 *p* = 0.08 for both subscales). These results imply that couples who completed the conflict prevention program had improved their ability to perceive negative situations in their relationship to be more a result of natural differences than intentional efforts to hurt one another and to contemplate relationship problems from a more descriptive and objective (i.e., less judgmental) perspective. The control group showed no significant nor near-significant differences in *IBCTQ* variables during the experimental group’s intervention period. 

For the *ASPA-Form A*, neither the experimental nor the control group showed significant differences in communication type (assertive, aggressive, submissive, passive-aggressive) pre and posttreatment. 

At the three-year follow-up, the experimental group’s average scores for the *DAS* and *IBCTQ* were very similar to their post-intervention values with no significant changes in *Z* score, implying the maintenance of intervention gains during the three years, although the dispersion for dyadic adjustment was large, suggesting caution regarding that value. The control group saw no significant changes in their *DAS* and *IBCTQ* scores during the follow-up period. They did show several near-significant changes in these variables, but all in a negative direction, possibly indicating a small decline in relationship quality. Interestingly, they also demonstrated a significant uptick in passive-aggressive communication on the *ASPA-Form A* at three-year follow-up, which would corroborate the hypothesis regarding relationship quality decline.

In order to highlight the existence of differences between the experimental and control group, a variable was created to reflect the changes between pre- and post-intervention data, eliminating the intrasubject component. Diverse comparisons were carried out using Mann–Whitney’s *U* in a process similar to a post hoc ANOVA. Table 4 displays these results.

Greater changes were observed in the experimental group for each *DAS* dimension, with the greatest difference being in dyadic satisfaction. Comparing means with Mann–Whitney’s *U* revealed statistically significant differences in dyadic satisfaction (*U* = 1.000; *Z* = −2.766; *p* = 0.00) and dyadic adjustment (*U* = 5.500; *Z* = −2.009; *p* = 0.02). The experimental group also showed greater change in all *IBCTQ* variables, with statistically significant differences versus the control group for acceptance (*U* = 6.500; *Z* = −1.848; *p* = 0.03), empathic joining (*U* = 5.500; *Z* = −2.009; *p* = 0.02), unified detachment (*U* = 1.000; *Z* = −2.732; *p* = 0.00), and total *IBCTQ* score (*U* = 6.000; *Z* = −1.925; *p* = 0.03), with empathic joining showing the greatest difference between groups. No significant differences were found in the analysis of changes at follow-up, again indicating the maintenance of intervention gains. 

With regard to the *ASPA-Form A*, no significant differences were found between the experimental and control groups. 

Analysis of qualitative data revealed satisfaction and a positive evaluation of the conflict prevention program by program participants. In accordance with the 5-point scale established to measure subjective perception of different aspects of the program, members of the experimental group had the following mean scores: general satisfaction regarding the individual’s expectations for the program—3.8; interest in the topics covered—4; interest in the strategies presented—3.8; possibility of applying these strategies—3.6; perception that these strategies would be helpful for addressing future marital problems—3.3; level of satisfaction regarding the professional who had imparted the program—4; and recommendation of the program to other couples—4. General satisfaction with the program received was 26.33 out of a 28-point maximum possible score.

In-session notes of experimental couples’ verbalizations seem to note changes in their patterns of interaction as a result of the intervention program. In the first session, comments tended to focus on couples’ desire to prevent or better manage conflict: “Being able to prevent discussions caught my attention”; “If we would do something before [things get out of hand], that would be great”; “We tend to have big arguments from time to time and I would like to know what to do”; “Sometimes I’m badly affected when we fight”. By the second session, couples were starting to be more aware of their differences and the tendency to polarize: “This week, I’ve focused more on our differences”; “I’ve focused more on the differences that annoy me”; “I see that we have a lot of differences”; “In a discussion we had on Saturday, each of us became rigid in their point of view”; It’s difficult to get outside of your point of view when you’re emotionally altered, but having talked about it last session at least it makes you think”. The third session showed greater awareness of spontaneous conflict resolution techniques that were functional for the couple: “We complement each other well; each one knows how to calm the other”; “This week when my partner got angry, I went to give them a hug”; “I can’t deny him a hug when he’s altered, that’s how we resolve things”; “We looked at the most useful way in which we deal with conflict: humor”. In the fourth session, couples began to comment on the tendency to attack each other instead of expressing soft emotions and their efforts to implement empathic joining: “I used last week’s strategy without having to think about it”; “The psychologist told us that we could express ourselves without attacking each other”; “It’s difficult to not attack each other when we’re emotionally altered, but it’s true that it complicates things”; “The other day I saw how we got stuck in a vicious circle because of this”; “I became aware of how much we criticize each other underneath everything.” In the fifth session, couples mentioned the helpfulness of analyzing problems more objectively as a team through unified detachment: “We tend to try and see problems objectively”; “I really liked what you said, that if a one of us has a problem and it affects both of us, then it’s our problem together”; “It’s logical that if we look at problems as a team it will be easier to resolve them”; “Being a team is very important”; “We’ve stopped somewhat being [a team] since a while ago”; “I also [believe] that if you’re not [a team], then what are you?”. They also emphasized the importance of tolerance strategies such as self-care: “I also look to my needs”; “Yes, when we’ve had an argument, I know that things will be okay if I dedicate time to be with friends and practice a sport and calm myself”; “Maybe we’ve lost time for ourselves [as individuals].” All of these comments are coherent with IBCT strategies (empathic joining, unified detachment, and tolerance) and reveal progress in the program’s objectives. 

Not all couples responded equally to all program elements. Couples showed more interest in and benefited more from specific IBCT strategies as a function of their interaction profile. For example, Experimental Couple 1 (EC1) responded more to unified detachment. They were the youngest of the cohort, had been together the shortest period of time, did not live together, and were memorable because of each partner’s openness in expressing emotions and their attempts to attend to each other’s feelings during conflict. Perhaps this openness was the result of cultural influences given the greater gender equality lived by current Spanish youth. Nevertheless, they tended to become tied up in these emotions and suffer growing distress because of the guilt each felt for the emotional pain they were causing in the other. At the same time, they tended to fuse with the content of their conflict, blaming each other for their difficulties instead of seeing the conflict as an external pattern or event that they fall into together. This tended to erode their sense of facing their problems as a team, once again increasing their level of distress. Unified detachment, a more analytic IBCT technique, helps to increase intimacy and positive affect toward the partner by promoting psychological distance from the problem, thus avoiding the blame game and supporting a more detached, “us vs. the conflict” mentality, all of which helped this couple to better navigate conflict. 

Experimental Couple 2, on the other hand, responded better to empathic joining. They were a middle-aged couple that had been together longer than EC1 and lived together without any children. They tended to take a logical approach to the problems that occurred between them, and when they expressed emotions, they would accuse each other vehemently, resulting in numerous conflicts and emotional distance from their partner and from themselves. They even understood emotions as problems to be solved. Empathic joining, which also looks to increase intimacy and positive affect toward one’s partner, helped them to directly and assertively express both their surface and hidden emotions as well as learn how to interpret their partner’s emotions by taking into account their behavioral learning history. They began to stop seeing emotions as a problem that needed to be solved. 

Lastly, Experimental Couple 3 responded the most to tolerance strategies. They had been together for many years, had two children, and showed a manifest resistance to change. They were aware of this resistance and explained that their many years together and the prior unfruitful attempts that they had made to change each other were important reasons for it. Thus, they showed the greatest interest and inclination toward tolerance strategies, especially being able to see the positive aspects of each partner’s negative behavior and the emphasis on self-care, the latter of which they had tried before but out of resignation instead of seeing it as a way of tolerating and accepting the other.

As can be seen through this qualitative analysis, each couple responded more to certain IBCT strategies in accordance with their personalities and relationship characteristics. Nevertheless, all showed quantifiable improvement on the *DAS* and *IBCTQ*, and several of these improvements were maintained over three years. It could be concluded that IBCT contains diverse strategies that benefit each couple in keeping with their specific relationship characteristics, but that there are also common factors that seem to benefit most or all couples. These common factors include: (1) an open and uninterrupted expression of the unpleasant emotions experienced by each partner during and after a conflict; (2) active listening by each partner in response to the emotions of the other; (3) consciousness raising between partners regarding each one’s learning history; (4) a more careful and assertive use of language when revealing emotions, putting emphasis on expressing feelings instead of criticism; (5) achieving distance from problems, seeing them from a more objective perspective, and (6) greater introspection and critical reflection toward the behaviors used by each to manage unpleasant emotions.

## 4. Discussion

The present investigation presents a new IBCT-based conflict prevention program for couples and conducts a pre-pilot test of its short-term and long-term efficacy with an experimental and control group. In contrast with previous marriage prevention programs, a single theoretical base (IBCT) was used to develop program content, and both quantitative and qualitative measures were used to investigate multiple intervention effects in line with that theory (changes in level of acceptance, empathic joining, unified detachment, and tolerance). The study’s follow-up period (three years) is also longer than that of most other marriage prevention investigations. Results reveal that the program can be efficacious in increasing relationship quality in the short-term and long-term (although the dispersion of the results for dyadic adjustment suggest care regarding the interpretation of that particular value), with the logical caution imposed by the small sample size. More specifically, the program contributes to increases in relationship satisfaction (mostly short-term) and affective expression (both short and long-term), which translates as a decrease in tension and an increase in intimate and sexual communication between partners. Other dimensions of relationship quality such as cohesion and consensus (*DAS*) also improve in couples post-intervention, although such changes only reach near-significance. We hypothesize that a longer training may favor clearer statistical results in these areas. 

With regard to specific IBCT variables, this program helps couples develop a greater use of empathic joining and unified detachment (*IBCTQ*). These results suggest that couples who complete the program are better able to conceive negative situations and actions (or inaction) by their partner as examples of differences that exist in the couple instead of personal attacks, just as the IBCT model posits [21], which are results that continue to suggest that the IBCT model applies cross-culturally. Couples also seem to gain a greater distance or separation from their problems: that is, a more objective and descriptive perspective of them. Associated measures of acceptance and tolerance also show near-significant change. 

Although slight changes in communication types (assertive, aggressive, submissive, and passive-aggressive, *ASPA—Form A*) were observed, none were significant. One possible reason is that IBCT acceptance and tolerance strategies do not directly focus on couple communication types but rather approach them transversely.

Both independent (pre–post changes in the experimental and control groups) and comparative analyses indicate the greatest change in *IBCTQ* variables, which is congruent with the program’s focus. This result both confirms that the program’s content really has followed IBCT principles and that the *IBCTQ* is sensitive to changes in IBCT variables. This last finding is of additional interest, since the *IBCTQ* is a relatively recent instrument that could be used in the future to measure other IBCT-based interventions. Along this line, we believe that the results of studies such as Rogge et al. [20] might have been different or clearer in their conclusions if they had benefitted from a more specific instrument for measuring IBCT variables such as the *IBCTQ*. It is also possible that a more specific and in-depth focus on IBCT components in the current program contributed to the different results between studies.

Qualitative data tend to be more sensitive to subjective interpretation. Nevertheless, the results in this case are so clear and consistent among participants that there is little doubt concerning couples’ satisfaction with the program, that the intervention increased feelings of rapport and understanding between partners, and that participants perceived that they had acquired useful tools for managing future conflicts. Role-play practice of IBCT strategies and the therapists’ resulting feedback do not guarantee that empathic joining and other abilities were fully absorbed by couples. However, follow-up results seem promising, and the manifest satisfaction of the participants who learned them indicates their likely use.

Although the results of this study are encouraging, limitations exist that suggest caution in generalizing the results. Due to the small sample size of the experimental and control groups (*n* = 12), this investigation should be considered as more pre-pilot than final. In addition, as previously mentioned, one of the experimental couples did not complete the three year-follow up data, expressing that they had separated. While this might seem worrisome, other marriage prevention studies indicate that a significant number of couples drop out or separate despite initial intervention gains [23,26,27]. The couple that separated also had a shorter relationship (2 years) than most of the others and did not live together, which seem to indicate a lower level of commitment compared with other participants and make the separation less surprising. The study’s small sample size also makes statistical anomalies of this type more probable and is one of the reasons that a follow-up study with a greater sample size is recommended. There were also temporal limitations to the IBCT content that could be imparted. Since we did not want this program to exceed other proposals such as that of Rogge et al. [20] in sessions or time, content was presented in five 120-minute sessions, which restrained the extension and variety of material used. We believe that complementing the intervention with videos and content summaries would more clearly illustrate each IBCT strategy.

## 5. Conclusions

The present investigation has sought to correct some of the limitations of earlier relationship prevention studies by creating a conflict prevention program that (1) shows significant long-term positive effects, (2) possesses strict and well-defined content to help identify effective prevention components, and (3) is based on a single theoretically and experimentally sound model (Integrative Behavioral Couple Therapy). Results reveal that the program is efficacious in increasing relationship quality in the short-term, with some long-term benefits, especially in the area of affectional expression. IBCT-related variables (empathic joining and unified detachment) showed significant short-term change, which were maintained at three-year follow-up. Associated measures of acceptance and tolerance also showed near-significant change. These data suggest that couples who complete the program are better able to view negative situations and actions (or inaction) by their partner as examples of differences that exist in the couple instead of personal attacks and gain a greater distance or separation from their problems, just as the IBCT model posits [21]. Both expressed satisfaction levels, and the 100% program completion rate seem to indicate high tolerance for the intervention, and the quantitative improvements seem to indicate no iatrogenic effects. While the recruitment rate for the investigation was good (75% of couples that desired to participate met with inclusion criteria and entered the study), recruitment itself was weak, with only eight couples responding to initial social media propaganda. Greater recruitment time and additional recruitment methods will need to be taken into account when conducting a full-size randomized controlled trial. 

While only a pre-pilot study, we believe the present investigation demonstrates the potential that a purely IBCT-based model has for relationship prevention both short-term and long-term and recommend its testing in a larger sample. We hope that the divulgation of empirical studies such as this one will help alleviate the absence of long-term efficacious conflict prevention programs and help to better define the usefulness of specific intervention components (in our case, empathic joining and unified detachment) in avoiding the escalation of serious couple conflict and its grave repercussions.

## Figures and Tables

**Table 1:** Sociodemographic characteristics of participants (*n* = 12).

Characteristic	Category	*n* (%)
Sex	Men Women	6 (50) 6 (50)
Age, mean (SD)		39.08 (13.49)
Education level	Low (Primary–Secondary) Moderate (Bachiller–Some Trade School) High (University)	2 (16.66) 2 (16.66) 8 (66.66)
Civil status	Couple (no legal union) Married	4 (33.33) 8 (66.66)
Relationship duration	years 15 years 36–38 years	6 (50) 2 (16.66) 4 (33.33)
Previous romantic partners	None 1 2 3 5	4 (33.33) 3 (25) 3 (25) 1 (8.33) 1 (8.33)
Living together	Yes No	8 (66.66) 4 (33.33)
Number of children	0 2	8 (66.66) 4 (33.33)
Occupation	Working Student	8 (66.66) 4 (33.33)
Previously attended couple therapy	No	12 (100)

**Table 2:** Sessions, objectives, content, techniques, and homework for the IBCT-based conflict prevention program.

Session	Objectives	Content	Techniques	Homework
1	- To present the program. - To collect general data on the couple. - To explore the beginning of the couple’s relationship. - To describe the couple’s current relationship and concerns regarding it. - To facilitate the couple’s understanding of the origins of their conflicts.	- Explanation of the preventative, empirical nature of the program and its objectives. - Review of sociodemographic information. - Positive and negative aspects, and differences noticed at the beginning of the relationship. - Positive and negative aspects, and differences noticed currently in the relationship. - Couple’s concerns regarding their relationship. - Origins of couple conflicts according to IBCT.	- Semi-structured interview. - Psychoeducation. - Reinforcement of the behaviors on which IBCT is based. - Feedback at the end of the session.	- Completion of self-reports. - Documentation of the couple’s doubts and questions.
2	- To explore the couples’ feelings and appreciation of important relationship events since the last session. - To review the previous session’s content and resolve doubts and questions about it. - To learn about and identify the couples’ spontaneous solutions to conflict. - To resolve doubts and consolidate the session’s main therapeutic points.	- The couples’ feelings and appreciation of important relationship events since the last session. - Content shared in the previous session. - Discussion of the couples’ spontaneous solutions to conflict, and their: (1) fit with each other, (2) level of attraction, (3) personality styles, (4) use of conflict resolution skills, and (5) stressful situations they are facing.	- Unstructured interview. - Psychoeducation. - Reinforcement of the behaviors on which IBCT is based. - Feedback at the end of the session.	- Documentation of the couple’s doubts and questions.
3	- To explore the couples’ feelings and appreciation of important relationship events since the last session. - To review the previous session’s content and resolve doubts and questions. - To facilitate understanding and internalization of empathic joining as an acceptance strategy. - To resolve doubts and consolidate the session’s main therapeutic points.	- The couples’ feelings and appreciation of important relationship events since last session. - Content shared in the previous session. - Strategies that strengthen acceptance in the couple, part I: empathic joining. Focusing on: (1) definition, (2) implications, (3) what it looks like, and (4) how it is accomplished. The couple is asked to select a situation to which they can apply this strategy and practice it in session.	- Unstructured interview. - Psychoeducation. - Role playing. - Reinforcement of the behaviors on which IBCT is based. - Feedback at the end of the session.	- Documentation of the couple’s doubts and questions.
4	- To explore the couples’ feelings and appreciation of important relationship events since the last session. - To review the previous session’s content and resolve doubts and questions. - To facilitate understanding and internalization of unified detachment as an acceptance strategy. - To resolve doubts and consolidate the session’s main therapeutic points.	- The couples’ feelings and appreciation of important relationship events since last session. - Content shared in the previous session. - Strategies that strengthen acceptance in the couple, part II: unified detachment. Focusing on: (1) definition, (2) implications, (3) what it looks like, and (4) how it is accomplished. The couple is asked to select a situation to which they can apply this strategy and practice it in session.	- Unstructured interview. - Psychoeducation. - Role playing. - Reinforcement of the behaviors on which IBCT is based. - Feedback at the end of the session.	- Documentation of the couple’s doubts and questions.
5	- To explore the couples’ feelings and appreciation of important relationship events since the last session. - To review the previous session’s content and resolve doubts and questions. - To facilitate understanding and internalization of tolerance strategies. - To resolve doubts and consolidate the session’s main therapeutic points. - To consolidate content from all five sessions and to resolve any final doubts regarding the material.	- The couples’ feelings and appreciation of important relationship events since last session. - Content shared in the previous session. - Strategies that strengthen tolerance. Focusing on: (1) definitions, (2) implications, (3) what they look like, and (4) how they are accomplished. Strategies: (1) highlight the positive aspects of a negative behavior, (2) fake negative behaviors at home, (3) promote self-care. - Review of content from the entire program.	- Unstructured interview. - Psychoeducation. - Role playing. - Reinforcement of the behaviors on which IBCT is based. - Feedback at the end of the session.	- Completion of self-reports.

**Table 3:** Wilcoxon Z and associated probability (*p*) results pre–post and post-follow-up for experimental and control groups (*DAS*, *IBCTQ*, and *ASPA-A*).

			Experim. Gr.		Wilcoxon		Control Gr.		Wilcoxon	
Scale	Dimension	Moment	$\bar{X}\;$	*SD*	*Z*	*P*	$\bar{X}\;$	*SD*	*Z*	*p*
DAS	DC	Pre	46.67	3.141	−1.511 −0.182	0.06 0.85	54.50	4.324	−0.135 −1.992	0.44 0.50
		Post	48.67	4.502			54.33	3.830		
		3-year	49.25	12.527			41.00	9.230		
	DS	Pre	33.83	4.070	−2.032 −1.841	0.02 * 0.06	39.67	5.007	−1.890 −1.572	0.06 0.12
		Post	38.33	3.061			38.83	4.355		
		3-year	34.25	3.594			28.83	12.189		
	AE	Pre	7.00	2.098	−1.761 −1.000	0.03 * 0.32	9.17	1.329	0.000 −1.753	10.00 0.08
		Post	9.00	0.632			9.17	1.329		
		3-year	8.75	0.500			5.67	4.033		
	DCH	Pre	13.83	2.714	−1.355 −.368	0.08 0.71	17.33	5.274	−.447 −1.265	0.32 0.21
		Post	15.33	3.327			17.50	4.370		
		3-year	17.25	2.872			14.00	6.986		
	TOTAL	Pre	101.33	7.367	−1.897 −0.535	0.02 * 0.59	120.67	11.021	−0.542 −1.782	0.29 0.07
		Post	111.83	7.360			119.83	12.287		
		3-year	109.50	16.258			89.50	30.723		
IBCTQ	A	Pre	63.33	8.548	−1.363 −0.365	0.08 0.71	69.67	11.911	−0.742 −1.753	0.22 0.08
		Post	70.17	9.411			68.83	13.318		
		3-year	73.00	12.356			59.83	21.236		
	EJ	Pre	78.33	10.053	−1.782 −0.816	0.03 * 0.41	83.67	11.130	−0.850 −1.892	0.19 0.06
		Post	84.33	4.884			82.67	12.356		
		3-year	85.00	6.218			70.50	20.197		
	UD	Pre	36.83	11.907	−2.201 −1.289	0.01 * 0.19	46.83	7.705	−1.134 −1.682	0.12 0.09
		Post	45.50	8.142			47.50	7.662		
		3-year	46.00	6.218			42.17	12.922		
	T	Pre	55.67	23.367	−1.363 −0.365	0.08 0.71	61.50	4.506	−0.841 −0.946	0.20 0.34
		Post	62.00	10.770			62.50	8.019		
		3-year	63.00	6.055			59.00	11.832		
	TOTAL	Pre	234.14	37.032	−1.997 0.000	0.02 * 10.0	261.67	33.827	−0.542 −1.572	0.30 0.12
		Post	262.00	24.658			261.50	39.773		
		3-year	267.00	25.626			231.50	65.056		
ASPA-A	CAs	Pre	45.17	8.909	−1.843 −0.730	0.06 0.46	53.00	5.733	−0.736 −0.674	0.23 0.5
		Post	48.83	5.419			52.33	4.131		
		3-year	45.25	7.805			49.00	9.529		
	CAg	Pre	23.17	9.600	−1.089 −1.461	0.13 0.14	23.50	9.854	0.000 −1.153	0.50 0.25
		Post	18.50	5.505			23.50	10.407		
		3-year	16.25	3.594			30.83	16.376		
	CS	Pre	23.17	10.610	−.315 −0.365	0.37 0.71	15.17	3.710	−0.946 −1.363	0.17 0.17
		Post	24.17	9.196			17.50	6.317		
		3-year	25.75	13.150			23.17	8.612		
	CPA	Pre	24.67	7.488	−0.962 −1.604	0.16 0.11	20.50	10.193	−1.633 −2.023	0.06 0.04 *
		Post	22.67	8.287			21.50	9.935		
		3-year	21.00	6.976			31.83	14.932		

* *p* ≤ 0.05. Note: DAS = Dyadic Adjustment Scale, DC = Dyadic Consensus, DS = Dyadic Satisfaction, AE = Affectional Expression, DCH = Dyadic Cohesion; IBCTQ = Integrative Behavioral Couple Therapy Questionnaire, A = Acceptance, EJ = Empathic Joining, UD = Unified Detachment, T = Tolerance; ASPA-A = Couple Assertiveness Questionnaire—Form A, CAs = Assertive Communication, CAg = Aggressive Communication, CS = Submissive Communication, CPA = Passive-Aggressive Communication.

**Table 4:** Mann–Whitney, Z, and associated probability (*p*) results comparing pre–post and post-follow-up changes between experimental and control groups.

			PRE–POST Comparison				POST-FOLLOW-UP Comparison			
Scale	Dimen.	Group	Average Rank	M-W *U*	*Z*	*p*	Average Rank	M-W *U*	*Z*	*p*
DAS	DC	Exper.	7.75	10.50	−1.21	0.11	4.13	6.50	−1.18	0.257
		Contr.	5.25				6.42			
	SD	Exper.	9.33	1.00	−2.76	0.00 *	4.00	6.00	−1.29	0.257
		Contr.	3.67				6.50			
	AE	Exper.	8.00	9.00	−1.61	0.05	3.25	3.00	−1.98	0.067
		Contr.	5.00				7.00			
	DCH	Exper.	7.67	11.00	−1.15	0.12	5.50	12.00	0.00	10.00
		Contr.	5.33				5.50			
	TOTAL	Exper.	8.58	5.50	−2.09	0.02 *	3.88	5.50	−1.39	0.171
		Contr.	4.42				6.58			
IBCTQ	A	Exper.	7.75	6.50	−1.85	0.03 *	5.63	11.50	−0.11	0.914
		Contr.	7.25				5.42			
	EJ	Exper.	9.33	5.50	−2.01	0.02 *	3.75	5.00	−1.50	0.171
		Contr.	3.67				6.67			
	UD	Exper.	8.00	1.00	−2.73	0.00 *	5.25	11.00	−0.21	0.914
		Contr.	5.00				5.67			
	T	Exper.	7.67	12.50	−0.88	0.18	4.88	9.50	−0.53	0.610
		Contr.	5.33				5.92			
	TOTAL	Exper.	8.33	6.00	−1.93	0.02 *	4.63	8.50	−0.74	0.476
		Contr.	4.42				6.08			
ASPA-A	CAs	Exper.	8.08	8.50	−1.55	0.06	6.88	6.50	−1.17	0.257
		Contr.	4.92				4.58			
	CAg	Exper.	5.92	14.50	−0.56	0.20	5.00	10.00	−0.43	0.762
		Contr.	7.08				5.83			
	CS	Exper.	6.42	17.50	−0.08	0.4	6.63	7.50	−0.96	0.352
		Contr.	6.58				4.75			
	CPA	Exper.	5.67	13.00	−0.82	0.2	4.38	7.50	−0.96	0.352
		Contr.	7.33				6.25			

* *p* ≤ 0.05. Note: DAS = Dyadic Adjustment Scale, DC = Dyadic Consensus, DS = Dyadic Satisfaction, AE = Affectional Expression, DCH = Dyadic Cohesion; IBCTQ = Integrative Behavioral Couple Therapy Questionnaire, A = Acceptance, EJ = Empathic Joining, UD = Unified Detachment, T = Tolerance; ASPA-A = Couple Assertiveness Questionnaire—Form A, CAs = Assertive Communication, CAg = Aggressive Communication, CS = Submissive Communication, CPA = Passive-Aggressive Communication.

## Data Availability

The data presented in this study are available on request from the corresponding author. The data are not publicly available due to privacy and ethical reasons.
